# Numerical Feedback Roundness Affects the Choice of the Self vs. Others as a Reference Point

**DOI:** 10.3389/fpsyg.2021.758990

**Published:** 2021-11-23

**Authors:** Meyrav Shoham, Nira Munichor

**Affiliations:** ^1^Dan Department of Communication, Tel Aviv University, Tel Aviv-Yafo, Israel; ^2^The Graduate School of Business Administration, Bar-Ilan University, Ramat-Gan, Israel

**Keywords:** reference points, numerical information, numerical roundness, temporal comparisons, social comparisons, self-evaluation, feedback

## Abstract

People can use social or personal information as a reference point against which they compare their performance. While previous research has shown that reference point choice can be affected by individual characteristics, situational factors, and goals, we suggest that properties of the performance feedback itself can also play a role in this choice. We focus on the effects of round vs. precise numerical feedback on reference point preferences. In three studies, we show that people are more likely to use themselves as a reference point to evaluate their performance following a feedback in the form of a round score (e.g., a score of 70 in a task) and to use others as a reference point following a precise score (e.g., a score of 71). Study 1 shows decreased interest in comparisons with others following round rather than precise feedback. Study 2 shows that round (vs. precise) feedback also increases actual choice of the self (vs. others) as a reference point. Study 3 demonstrates that the effect of the numerical feedback on reference point preferences extends to the choice of a benchmark for future comparisons. We discuss the implications of our results for the literature and practice, including how this can be used to encourage desirable behaviors.

## Introduction

Imagine downloading a trivia app and taking a couple of quizzes. How would you determine how well you did? One possibility is to find out how other app users performed on the quiz, and compare your achievement to theirs; another would be to focus on your own progress and achievements. Would a round vs. a precise quiz score affect your choice of reference point?

People can evaluate themselves based on feedback that they receive regarding their skills, performance, and actions (Ashford, 1986; Ashford and Tsui, 1991; Anseel et al., 2007). To interpret the feedback, people can rely on objective information. They can also perform social comparisons, gauging their performance by using others as a reference point (Festinger, 1954; Suls and Miller, 1977; Suls and Wills, 1991; Moore and Klein, 2008), or use themselves as a reference point and compare their current performance to an earlier one (Albert, 1977; Wilson and Ross, 2001). We suggest that the roundness vs. precision of a numerical feedback (e.g., a score of 70 vs. a score of 71) affects the choice of a reference point.

## Theoretical Background and Hypotheses Development

### Reference Points for Feedback Interpretation: Others vs. Oneself

Feedback plays an important role in self-evaluation processes, providing information about where one stands and how one is doing with respect to a pursued goal (Ashford and Tsui, 1991; Anseel et al., 2007). For example, students use test scores to get feedback on their achievements and learning outcomes (Wisniewski et al., 2020), and employees seek feedback on their work performance (De Stobbeleir et al., 2011; Su et al., 2019).

At times, the received feedback allows for objective testing of one’s performance (e.g., Klein, 2003). Sometimes, however, feedback alone cannot provide a clear test of one’s abilities (Festinger, 1954). For example, the number of items completed in an intelligence test does not fully capture the test taker’s intelligence. In such cases, comparing the feedback against a reference point can aid in its interpretation (Festinger, 1954; Albert, 1977). Even when the feedback is sufficient for assessment, people may be interested in a relative perspective (Miller, 1977; Klein, 1997). Both information about the self and about other people can serve as a reference point against which the received feedback is compared.

Festinger’s (1954) seminal social comparison theory views other people as a reference point against which people evaluate themselves. This can be rewarding if one’s social comparison status is above average and if it is increasing over time (Zell and Alicke, 2010). The influence of social reference points on self-evaluation is apparent in a broad range of situations ranging from a person’s relative position in a queue (Buell, 2020) to comparisons to attractive others (Cattarin et al., 2000). Information about others’ performance can also affect the way people approach certain tasks and their willingness to take risks (Wang et al., 2016).

Sometimes, however, people forego social comparisons and use themselves as a comparison standard. People can assess their performance based on their personal standards, namely, their expectations or goals (Wayment and Taylor, 1995) or their perceived self-potential (Markus and Nurius, 1986). In Albert’s (1977) temporal comparison theory, people can compare their present standing to their past ones. These self-temporal comparisons help maintain a sense of identity over time and adjust to perceived changes of the self; self-temporal comparisons produced a sense of progress among children and made them feel proud of themselves (Gürel et al., 2020).

Albert (1977, p. 490) suggested that information about other people, if available, will be preferred as a reference point for self-evaluation over information about the self (see also Festinger, 1954). More recently, Zell and Strickhouser (2020) found that social comparisons exert a significantly greater effect on self-evaluations than temporal comparisons.

Nonetheless, reference point preference can vary. Situational factors can affect the choice of a reference point; for example, stress and uncertainty often facilitate social comparisons (Molleman et al., 1986; Taylor et al., 1990; Gibbons and Buunk, 1999). The specific goal of a self-evaluation process can also affect reference point preference. Self-temporal comparisons can be rewarding due to skills improving over time or because past performance can be relatively easily criticized and disparaged. Thus, self-comparisons may be more likely to serve self-enhancement goals (Wilson and Ross, 2001; Zell and Alicke, 2009). Social comparisons, meanwhile, may be more appropriate when people want to obtain accurate self-related information (Wilson and Ross, 2000). Personal characteristics can also affect the choice of a reference point. High self-esteem, for instance, increases reliance on the self as a source of comparison (Wayment and Taylor, 1995) as do very young or old ages (Kulik and Ambrose, 1992). In this paper, we investigate factors related not to the circumstances, the person or the goal of the self-evaluation process, but to the properties of the feedback itself, namely, the precision vs. roundness of a numerical feedback.

### Effects of Round vs. Precise Numbers

People encounter numerical information in various contexts in everyday life. Evaluations and judgments affected by the properties of numerical information include financial decision-making (Isaac et al., 2020), donation behavior (Ye et al., 2021), behavior in negotiations (Mason et al., 2013), goal pursuit (Munichor and LeBoeuf, 2018), and product evaluations (Santana et al., 2020). A robust finding is that people perceive and react differently to round vs. precise numbers. Round numbers are those that end with more zeroes, while precise numbers end in fewer zeroes or no zeroes (Thomas et al., 2010). Inferences about round and precise numbers can affect a range of perceptions and behavioral outcomes.

Precise numbers often draw people’s attention and are evaluated as credible and accurate (Santos et al., 1994; Schindler and Yalch, 2006; Xie and Kronrod, 2012). Negotiators who use precise numbers in their offers come across as better informed, leading to counteroffers closer to the amount they had named (Janiszewski and Uy, 2008; Mason et al., 2013). Zhang and Schwarz (2012, 2013) suggested that people assume that a communicator uses a precise number for a reason. Because social comparisons are perceived as informative, people could try to understand what, exactly, a precise numerical feedback represents by checking where it stands compared to other people’s scores.

Round numbers, meanwhile, can denote completion and stability. Thus, people perceive energy drinks and pills that come in round-number doses as more effective than those whose volume was precise (Pena-Marin and Bhargave, 2016), and negotiators are more willing to accept round offers than comparable precise offers (Yan and Pena-Marin, 2017). People are also more likely to select retirement investments targeted to retirement at a round (vs. precise) age (Kalra et al., 2020) and to pay off round-number debts (Isaac et al., 2020).

Round numbers can be seen as perceptual boundaries between numerical categories, and people view changes that cross into a new category as more meaningful (Isaac and Schindler, 2014; Shoham et al., 2018). This is reflected in goal-driven behavior: People strive to reach round thresholds such as a sub-4-h marathon or a round SAT score (Pope and Simonsohn, 2011; Allen et al., 2017). A round-number performance may therefore be seen as personally meaningful. This can encourage people to reflect on their own progress and achievements (Wilson and Ross, 2001). Some support for this idea can be found in Alter and Hershfield’s (2014) research, which showed that nearing a round age milestone inspires self-reflection and assessment of one’s progress.

In sum, the different perceptions of round vs. precise numbers suggest that the roundness of numerical feedback should affect the choice of a reference point against which people assess feedback and evaluate their performance. Round numbers can serve as personally meaningful reference points and consequently spur people to contrast their past and future selves (Dai et al., 2014). Consequently, round numerical feedback should motivate people to take a greater interest in what they achieved in the past. Thus:

*H1*: Receiving a round feedback score for one’s performance will encourage temporal self-comparisons.

People assume that precise information is used for a reason and conveys meaningful subtleties (Zhang and Schwarz, 2012, 2013). Therefore, precise numerical feedback should lead people to try to understand exactly what the score represents in terms of achievement. Social comparisons can serve as relatively accurate benchmark that helps understand where one stands in terms of performance (Festinger, 1954). Thus:

*H2*: Receiving a precise feedback score for one’s performance will encourage social comparisons.

## Materials and Methods

Three experiments provide support for the effect of the roundness vs. precision of numerical feedback on reference point preference and choice. Our experiments employ different feedback score levels to explore the prevalence of the hypothesized effect. This approach is based on empirical considerations, in order to align our work with previous research that examined effects of numerical roundness and precision using several different values (Pope and Simonsohn, 2011; Tao et al., 2017; Hauser and Schwarz, 2019). We received IRB approval for the procedures.

The target sample size in our studies was selected based on previous research on the choice of reference points (e.g., Klein, 2003; Zell and Alicke, 2009) and numerosity (e.g., Xie and Kronrod, 2012; Zhang and Schwarz, 2012). Power analyses *via* G^*^Power confirmed that our target sample sizes provided adequate power to detect medium-sized effects (Faul et al., 2009; Cohen, 2013; Bosco et al., 2015). We excluded participants whose study completion time was extremely long (suggesting a possible lack of attention or focus).

### Study 1: The Effect of Numerical Feedback on Interest in Comparisons to Others

#### Participants

Two hundred and fifteen participants (57.2% female; *M*_age_=32.76) from the Prolific Academic participant pool took part in the study in exchange for monetary compensation. We randomly assigned participants to one of four conditions in a 2 (feedback type: round score vs. precise score)×2 (feedback score: high vs. moderately high)^1^ between-subjects design. We manipulated feedback score to test the robustness of the effect across different score levels.

#### Procedure

Participants completed two rounds of a word identification task and were told that they would be scored based on the accuracy and speed of their answers. Each round included ten lists of eleven words, revolving around a similar theme (e.g., marine animals). The lists were presented one at a time for 2s each. Participants then saw a word and were asked to indicate whether that word had appeared on the list. Following the first round, participants were reminded of the task instructions before proceeding to round two. After completion of the second round, participants were shown one of four scores as their “second round score”: 80 (round moderate score condition), 81 (precise moderate score condition), 90 (round high score condition), or 91 (precise high score condition). They then indicated (1=“Not at all” to 7=“Very much”) their interest in comparing their scores to that of other participants (“*How curious are you about your performance on this quiz compared to other people’s performance*”) and to their own previous score (“*How curious are you about your performance on this quiz compared to your performance in the first quiz*”). Finally, participants completed several demographic measures and were debriefed.

#### Results and Discussion

A two-factor MANOVA revealed a significant main effect for feedback type on curiosity about others’ performance. Participants who received a precise score in the second round were more curious about the performance of others in the task (*M*=5.79, *SD*=1.47) than participants who received a round score [*M*=5.35, *SD*=1.65; *F*(1, 211)=4.34, *p*=0.038].

The effects of feedback score (high or moderate) and the interaction between feedback score and feedback type on curiosity about others’ performance were not significant [*F*(1, 211)=0.41, *p*=0.521 and *F*(1, 211)=0.08, *p*=0.783, respectively]. Turning to curiosity about one’s past performance, we found no significant effect for feedback type [*F*(1, 211)=2.52, *p*=0.114], feedback score [*F*(1, 211)=1.30, *p*=0.255] or the interaction between them [*F*(1, 211)=0.60, *p*=0.439].

Study 1 provides initial evidence that the roundness of a numerical feedback may shift the preferred reference point for comparison. People are less interested in using others as a reference point when they receive a round numerical feedback as opposed to a precise one. This pattern was not affected by whether the score was very high or only moderately so, pointing to the robustness of the observed effect. Score roundness did not affect curiosity about one’s own previous score, possibly because *curiosity* is not a direct measure of preference. To address this, Study 2 examined the effect of feedback roundness on actual choice of a reference point.

### Study 2: Choice of Reference Point as a Function of Numerical Feedback Roundness

Study 2 examined people’s preferences by giving participants a choice between others and the self as a reference point. To demonstrate the robustness of the effect, we used a new task and precise numbers ending in digits other than 1. We also used two different levels of precise feedback, one higher and one lower than the round feedback. This was done to ensure that it is numerical roundness, rather than receiving a lower score that drives the preference for self-temporal comparisons.

#### Participants

One hundred and eighty participants (59% female, *M*_age_=34.78) from Prolific Academic took part in the study in exchange for monetary compensation. Participants were randomly assigned to one of three between-subjects conditions (round score vs. lower precise score vs. higher precise score).

#### Procedure

Participants completed two rounds of a trivia quiz, with ten multiple-choice questions in each round (e.g., “*Who was the first man to walk on the moon*?”). They were told that the score for each quiz would be calculated based on the number of correct answers and the time spent completing the quiz. This made intuitive sense and was also intended to prevent participants from searching for correct answers online and calculating their own scores. Following the first round, participants were reminded of the task instructions before proceeding to round two. Once they had completed the second round of the quiz, participants read that their score for that round was 80 (round score condition), 77 (lower precise score condition), or 83 (higher precise score condition). We then asked participants to rate their performance on the second quiz and told them that to help them do so, they could choose to see either their first round score or the average score obtained by other people in the second round of the quiz. Due to technical reasons, participants who chose to see their own previous score read that it was 75, and participants who chose others’ average score read that it was 73. In keeping with the cover story, participants rated their performance on the second quiz (1=“Extremely bad” to 7=“Extremely good”). Finally, participants completed demographic measures and were debriefed.

#### Results and Discussion

A logistic regression revealed no difference based on whether the precise score was higher or lower than the round score on the type of information participants chose to receive [*χ*^2^ (1)=0.41, *p*=0.523]. We therefore collapsed our data across the two precise score conditions. Participants were more likely to choose to receive their previous score rather than other’s average score when their score was round (67.21%) than when it was precise [47.90%; *χ*^2^ (1)=6.06, *p*=0.014]. Feedback roundness did not affect performance ratings [*M*_round_=4.89, *SD*_round_=1.13 vs. *M*_precise_=5.10, *SD*_precise_=0.91, *t*(178)=1.39, *p*=0.167].

Study 2 shows that the effect of feedback roundness extends to the actual choice of reference point. People who received a round score were less interested in using others as a reference point, and more focused on their past performance, than those who received a precise score. Because we used two different precise scores, the effect cannot be attributed to the round score being lower than the precise one, which might have led to concerns about others performing better and thus hindered self-enhancement (Wilson and Ross, 2001; Zell and Alicke, 2009).

### Study 3: Numerical Feedback, Choice of Reference Point, and Future Preferences

Study 3 provides further evidence for the robustness of the effect of feedback roundness on the choice of a reference point using a different task and more modest numerical feedback. This study also explores whether the effect extends to preferences for future comparisons.

#### Participants

One hundred and five participants (42.9% female, *M*_age_=32.51) from Prolific Academic took part in this study in exchange for monetary compensation. Participants were randomly assigned to one of two between-subjects conditions (round score vs. precise score).

#### Procedure

Participants took two consecutive geography quizzes with ten multiple-choice questions in each round (e.g., *Where is the Cape of Good Hope located?*). The procedure was otherwise similar to that of Study 2. After completing the second quiz, participants were shown a score of either 60 (round score condition) or 61 (precise score condition) as their “second round score.” We again asked participants to rate their performance on the second quiz and offered them a choice between their own score on the first quiz and others’ average score on the second quiz. The score shown was 55 regardless of their choice. In keeping with the cover story, participants then rated their performance on the second quiz (1=“Extremely bad” to 7=“Extremely good”). Next, they indicated how curious they would be about how well they would perform on a third quiz (a) compared to others and (b) compared to their own previous performance (1=“Not at all” to 7=“Very much”). Finally, participants completed demographic items and were debriefed.

#### Results and Discussion

**Figure 1:**
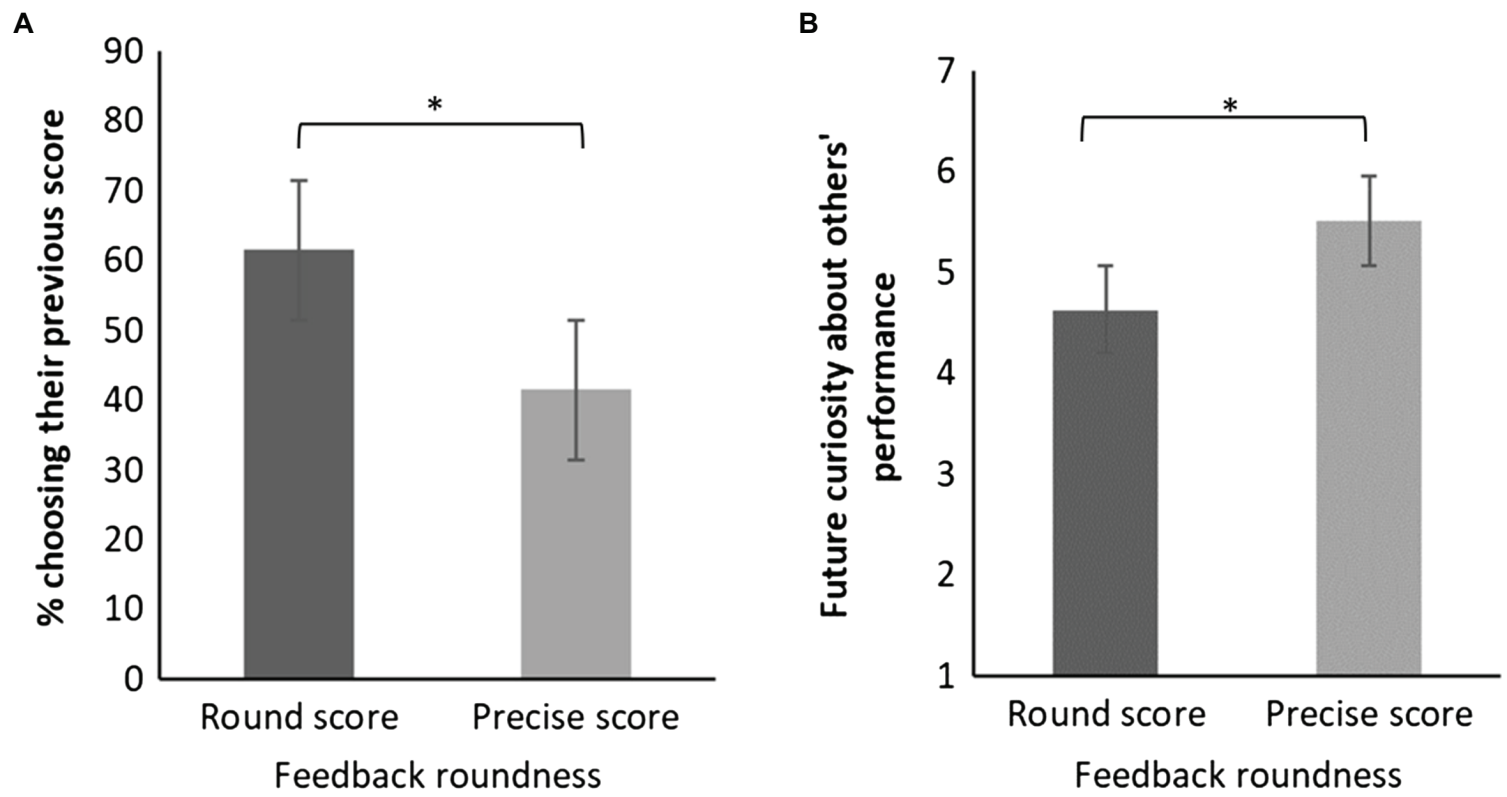
The effect of numerical feedback type on choice of feedback panel **(A)** and on future curiosity about others’ performance panel **(B)** in Study 3. **(A)** Error bars are standard error of the sample proportion. **(B)** Error bars are standard error of the sample mean. *p<0.05.

As predicted and shown in Figure 1, a majority (61.5%) of the participants who received a round score on the second task preferred to find out how they did on the first task. Among participants who received a precise score, the pattern was reversed, and the majority (58.5%) chose to compare their score to that of others [*χ*^2^ (1)=4.22, *p*=0.040].

In addition, participants who received a precise score in the second round were more curious about how they would perform in a future quiz compared to others (*M*=5.51, *SD*=1.40) than participants who received a round score [*M*=4.63, *SD*=2.09; *t*(103)=2.53, *p*=0.013]. We found no significant effect on curiosity relative to one’s past performance [*t*(103)=0.56, *p*=0.576]. Feedback roundness did not affect performance ratings [*M*_round_=4.33, *SD*_round_=1.49 vs. *M*_precise_=4.77, *SD*_precise_=1.35, *t*(103)=1.61, *p*=0.111].

Study 3 provides additional support for the preference for self-temporal rather than other-focused comparisons following round numerical feedback, using a lower score and a different task. In addition, Study 3 suggests that this effect may endure beyond the original task and affect preferences for evaluating future performance.

## General Discussion

People can rely on both social- and self-comparisons in order to interpret the numerical feedback they receive. We find that the roundness vs. precision of the feedback affects their choice of reference point for comparison. Receiving a precise (vs. round) score increases people’s interest in comparing their performance to that of other people, whether currently or in the future, and reduces the likelihood that they will opt for a self-temporal comparison. This effect is particularly prominent in actual choice behavior; influence on self-reported curiosity emerges with respect to others-focused, but not self-focused, comparisons. The feedback precision effect is robust across different tasks, different levels of numerical feedback, and occurs for precise numbers just above the round score, as well as those that are several units above or below it. Table 1 summarizes these results.

**Table 1:** Descriptive statistics for all studies.

Study/conditions	Curiosity about other’s scores *M* (SD)	Feedback choice: self (previous score)	Curiosity about future performance compared to others *M* (SD)
**Study 1**		n.a.	n.a.
Round feedback (80 or 90)	5.35 (1.65)				
Precise feedback (81 or 91)	5.79 (1.47)				

**Study 2**	n.a.		n.a.
Round feedback (80)				67.21%	
Precise feedback (77 or 83)				47.90%	

**Study 3**	n.a.		
Round feedback (60)			61.5%	4.63 (2.09)
Precise feedback (61)			41.5%	5.51 (1.40)

This research offers a new perspective on reference point preferences. Prior research on the selection between other people and oneself as a comparison target has focused primarily on effects of contextual factors (e.g., Taylor et al., 1990; Gibbons and Buunk, 1999), personal characteristics (e.g., Kulik and Ambrose, 1992; Wayment and Taylor, 1995), and personal goals (e.g., Wilson and Ross, 2001; Zell and Alicke, 2009). We extend this literature by showing that the roundness of a score also affects decisions about what benchmark to use in order to interpret that feedback.

Our findings shed further light on round numbers as reference points. Numbers can affect goal-driven behavior, with people striving to reach round-number goals (Pope and Simonsohn, 2011; Allen et al., 2017). We show that receiving a round number as *feedback* can affect the choice of additional reference points against which to evaluate one’s performance, encouraging self-focused comparisons. By doing so, this research provides a link between the literature on numerical roundness and that on personally meaningful reference points (Robinson, 1986; Shum, 1998; Thaler, 1999; Dai et al., 2014).

We also contribute to the broader literature on the effects of numerical roundness and precision. These characteristics of numerical information can affect how numbers are processed (Wadhwa and Zhang, 2015, 2019), how different targets are evaluated (Pena-Marin and Bhargave, 2016; Gunasti and Ozcan, 2019), and behavioral outcomes including financial decision-making (Isaac et al., 2020), responses to health messages (Wadhwa and Zhang, 2019), and consumer preferences (Santana et al., 2020). We show that numerical information can affect a novel outcome: whether to engage in self-temporal or other-focused comparisons.

Finally, we contribute to the literature on social and temporal comparisons. The traditional assumption is that people prefer to compare themselves to others when possible (Festinger, 1954). It has also been suggested that a focus on one’s own progress may serve self-enhancement goals (Wilson and Ross, 2001; Zell and Alicke, 2009), while social comparisons may be more appropriate when the goal is to obtain accurate self-related information (Wilson and Ross, 2000). We find a preference for temporal self-comparisons when information about the performance of others is also available, and there is no indication that the comparison would be self-enhancing.

A number of limitations and questions remain to be addressed. While we presented robust evidence for the effect, it remains to be directly tested whether a focus on the self vs. others (measured, e.g., through the Linguistic Implications Form; Wegner and Giuliano, 1980) mediates reference point choice. Furthermore, in order to manipulate numerical feedback cleanly and efficiently, we used a specific category of tasks (quizzes) that can be completed quickly, and we provided feedback indicating satisfactory performance for ethical reasons. Future research could examine whether the effect extends to other contexts in which people receive numerical feedback and to other score levels. Such research could also explore whether the effect of numerical feedback roundness shifts over time; a round score may have a stronger effect when it follows a more significant investment of time and effort. Preferences for temporal self-comparisons may also be more pronounced when people anticipate that their performance will be below average compared to others (Zell and Alicke, 2010).

Our findings open the door for future research on the downstream effects of reference point choice following numerical feedback. Such research could examine whether comparison to the self vs. others following round vs. precise numerical feedback affects the feedback receiver’s motivation and whether the individual’s motives (e.g., self-evaluation, self-enhancement, self-improvement; Suls et al., 2002) play a role in these effects.

Future research can also yield insights on whether numerical feedback precision can be used to facilitate desired behaviors. For example, prompting self-focused rather than others-focused comparisons may help instill pride without leading to feelings of superiority (Gürel et al., 2020). It could also help limit unwelcome phenomena brought about by social comparisons, such as cheating (John et al., 2014), by discouraging comparisons to others. Conversely, it may be possible to enhance task meaningfulness by adding a social dimension through precise feedback.

Numerical feedback is ubiquitous in people’s lives. They can encounter it at school, at work, and while engaging in hobbies and leisure activities. This feedback can lead to cognitive reactions and behavioral outcomes based on how the feedback is assessed. In this research, we show that the type of numerical feedback people receive can affect the choice of reference points against which they will compare their performance. Researchers and practitioners can benefit from understanding the implications of this reference point preference.

## Data Availability Statement

The raw data supporting the conclusions of this article will be made available by the authors, without undue reservation.

## Ethics Statement

The studies involving human participants were reviewed and approved by The Ethics Committee of Tel Aviv University. The patients/participants provided their written informed consent to participate in this study.

## Author Contributions

MS and NM designed the experiments, collected data, analyzed the data, and wrote the manuscript together. All authors contributed to the article and approved the submitted version.

## Conflict of Interest

The authors declare that the research was conducted in the absence of any commercial or financial relationships that could be construed as a potential conflict of interest.

## Publisher’s Note

All claims expressed in this article are solely those of the authors and do not necessarily represent those of their affiliated organizations, or those of the publisher, the editors and the reviewers. Any product that may be evaluated in this article, or claim that may be made by its manufacturer, is not guaranteed or endorsed by the publisher.

## Footnotes

^1^Based on typical grades in scholastic and academic settings in the United States of America (e.g., Gershenson, 2018); for brevity, we refer to these as high vs. moderate scores hereafter.

## References

[ref1] Albert S. (1977). Temporal comparison theory. Psychol. Rev. 84, 485–503. doi: 10.1037/0033-295X.84.6.485

[ref2] Allen E. J. Dechow P. M. Pope D. G. Wu G. (2017). Reference-dependent preferences: evidence from marathon runners. Manag. Sci. 63, 1657–1672. doi: 10.1287/mnsc.2015.2417

[ref3] Alter A. L. Hershfield H. E. (2014). People search for meaning when they approach a new decade in chronological age. P. Natl. A. Sci. 111, 17066–17070. doi: 10.1073/pnas.1415086111 PMC4260584 25404347

[ref4] Anseel F. Lievens F. Levy P. E. (2007). A self-motives perspective on feedback-seeking behavior: linking organizational behavior and social psychology research. Int. J. Manag. Rev. 9, 211–236. doi: 10.1111/j.1468-2370.2007.00210.x

[ref5] Ashford S. J. (1986). Feedback-seeking in individual adaptation: A resource perspective. Acad. Manag. J. 29, 465–487.

[ref6] Ashford S. J. Tsui A. S. (1991). Self-regulation for managerial effectiveness: The role of active feedback seeking. Acad. Manag. J. 34, 251–280.

[ref7] Bosco F. A. Aguinis H. Singh K. Field J. G. Pierce C. A. (2015). Correlational effect size benchmarks. J. Appl. Psychol. 100, 431–449. doi: 10.1037/a0038047, PMID: 25314367

[ref8] Buell R. W. (2020). Last-place aversion in queues. Manag. Sci. 67, 1430–1452. doi: 10.1287/mnsc.2020.3619

[ref9] Cattarin J. A. Thompson J. K. Thomas C. Williams R. (2000). Body image, mood, and televised images of attractiveness: The role of social comparison. J. Soc. Clin. Psychol. 19, 220–239. doi: 10.1521/jscp.2000.19.2.220

[ref10] Cohen J. (2013). Statistical Power Analysis for the Behavioral Sciences. New York, NY: Academic Press.

[ref11] Dai H. Milkman K. L. Riis J. (2014). Put your imperfections behind you: temporal landmarks spur goal initiation when they signal new beginnings. Psychol. Sci. 26, 1927–1936. doi: 10.1177/2F0956797615605818 PMC4839284 26546079

[ref12] De Stobbeleir K. E. Ashford S. J. Buyens D. (2011). Self-regulation of creativity at work: The role of feedback-seeking behavior in creative performance. Acad. Manag. J. 54, 811–831. doi: 10.5465/amj.2011.64870144

[ref13] Faul F. Erdfelder E. Buchner A. Lang A. G. (2009). Statistical power analyses using G^*^ power 3.1: tests for correlation and regression analyses. Behav. Res. Methods 41, 1149–1160. doi: 10.3758/BRM.41.4.1149, PMID: 19897823

[ref14] Festinger L. (1954). A theory of social comparison processes. Hum. Relat. 7, 117–140. doi: 10.1177/001872675400700202

[ref15] Gershenson S. (2018). Grade Inflation in High Schools (2005–2016). United States: Fordham Institute.

[ref16] Gibbons F. X. Buunk B. P. (1999). Individual differences in social comparison: development of a scale of social comparison orientation. J. Pers. Soc. Psychol. 76, 129–142. doi: 10.1037/0022-3514.76.1.129, PMID: 9972558

[ref17] Gunasti K. Ozcan T. (2019). The role of scale-induced round numbers and goal specificity on goal accomplishment perceptions. Market. Lett. 30, 207–217. doi: 10.1007/s11002-019-09492-w

[ref18] Gürel Ç. Brummelman E. Sedikides C. Overbeek G. (2020). Better than my past self: temporal comparison raises children’s pride without triggering superiority goals. J. Exp. Psychol. Gen. 149, 1554–1566. doi: 10.1037/xge0000733, PMID: 31944812

[ref19] Hauser R. Schwarz N. (2019). Score blending: how scale response grouping biases perceived standing. J. Behav. Decis. Making 32, 194–202. doi: 10.1002/bdm.2107

[ref20] Isaac M. S. Schindler R. M. (2014). The top-ten effect: consumers’ subjective categorization of ranked lists. J. Consum. Res. 40, 1181–1202. doi: 10.1086/674546

[ref21] Isaac M. S. Wang Y. Schindler R. M. (2020). The round-number advantage in consumer debt payoff. J. Consum. Psychol. 31, 240–262. doi: 10.1002/jcpy.1192

[ref22] Janiszewski C. Uy D. (2008). Precision of the anchor influences the amount of adjustment. Psychol. Sci. 19, 121–127. doi: 10.1111/j.1467-9280.2008.02057.x, PMID: 18271859

[ref23] John L. K. Loewenstein G. Rick S. I. (2014). Cheating more for less: upward social comparisons motivate the poorly compensated to cheat. Organ. Behav. Hum. Dec. 123, 101–109. doi: 10.1016/j.obhdp.2013.08.002

[ref24] Kalra A. Liu X. Zhang W. (2020). The zero bias in target retirement fund choice. J. Consum. Res. 47, 500–522. doi: 10.1093/jcr/ucaa035

[ref25] Klein W. M. (1997). Objective standards are not enough: affective, self-evaluative, and behavioral responses to social comparison information. J. Pers. Soc. Psychol. 72, 763–774. doi: 10.1037/0022-3514.72.4.763, PMID: 9108694

[ref26] Klein W. M. (2003). Effects of objective feedback and “single other” or “average other” social comparison feedback on performance judgments and helping behavior. Pers. Soc. Psychol. B. 29, 418–429. doi: 10.1177/0146167203251195 15273018

[ref27] Kulik C. T. Ambrose M. L. (1992). Personal and situational determinants of referent choice. Acad. Manag. Rev. 17, 212–237. doi: 10.5465/amr.1992.4279534

[ref28] Markus H. Nurius P. (1986). Possible selves. Am. Psychol. 41, 954–969. doi: 10.1037/0003-066X.41.9.954

[ref29] Mason M. F. Lee A. J. Wiley E. A. Ames D. R. (2013). Precise offers are potent anchors: conciliatory counteroffers and attributions of knowledge in negotiations. J. Exp. Soc. Psychol. 49, 759–763. doi: 10.1016/j.jesp.2013.02.012

[ref30] Miller R. L. (1977). Preferences for social vs. non-social comparison as a means of self-evaluation. J. Pers. 45, 343–355. doi: 10.1111/j.1467-6494.1977.tb00157.x, PMID: 894469

[ref31] Molleman E. Pruyn J. Van Knippenberg A. (1986). Social comparison processes among cancer patients. Brit. J. Soc. Psychol. 25, 1–13. doi: 10.1111/j.2044-8309.1986.tb00695.x 3947800

[ref32] Moore D. A. Klein W. M. (2008). Use of absolute and comparative performance feedback in absolute and comparative judgments and decisions. Organ. Behav. Hum. Dec. 107, 60–74. doi: 10.1016/j.obhdp.2008.02.005

[ref33] Munichor N. LeBoeuf R. A. (2018). The influence of time-interval descriptions on goal-pursuit decisions. J. Marketing Res. 55, 291–303. doi: 10.1509/jmr.14.0088

[ref34] Pena-Marin J. Bhargave R. (2016). Lasting performance: round numbers activate associations of stability and increase perceived length of product benefits. J. Consum. Psychol. 26, 410–416. doi: 10.1016/j.jcps.2015.11.004

[ref35] Pope D. Simonsohn U. (2011). Round numbers as goals: evidence from baseball, SAT takers, and the lab. Psychol. Sci. 22, 71–79. doi: 10.1177/0956797610391098, PMID: 21148460

[ref36] Robinson J. A. (1986). “Temporal reference systems and autobiographical memory,” in Autobiographical Memory. ed. Rubin D. C. (Cambridge: Cambridge University Press), 159–188.

[ref37] Santana S. Thomas M. Morwitz V. G. (2020). The role of numbers in the customer journey. J. Retailing 96, 138–154. doi: 10.1016/j.jretai.2019.09.005

[ref38] Santos M. D. Leve C. Pratkanis A. R. (1994). Hey buddy, can you spare seventeen cents? Mindful persuasion and the pique technique. J. Appl. Soc. Psychol. 24, 755–764. doi: 10.1111/j.1559-1816.1994.tb00610.x

[ref39] Schindler R. M. Yalch R. F. (2006). “It seems factual, but is it? Effects of using sharp vs. round numbers in advertising claims,” in Advances in Consumer Research. *Vol*. 33. eds. Pechmann C. Price L. L. (Duluth, MN: Association for Consumer Research), 586–590.

[ref40] Shoham M. Moldovan S. Steinhart Y. (2018). Mind the gap: how smaller numerical differences can increase product attractiveness. J. Consum. Res. 45, 761–774. doi: 10.1093/jcr/ucy022

[ref41] Shum M. S. (1998). The role of temporal landmarks in autobiographical memory processes. Psychol. Bull. 124, 423–442. doi: 10.1037/0033-2909.124.3.423, PMID: 9849113

[ref42] Su W. Lin X. Ding H. (2019). The influence of supervisor developmental feedback on employee innovative behavior: a moderated mediation model. Front. Psychol. 10:1581. doi: 10.3389/fpsyg.2019.01581 31338055 PMC6629885

[ref43] Suls J. M. Martin R. Wheeler L. (2002). Social comparison: why, with who, and with what effect? Curr. Dir. Psychol. Sci. 11, 159–163. doi: 10.1111/1467-8721.00191

[ref44] Suls J. M. Miller R. L. (1977). Social Comparison Processes: Theoretical and Empirical Perspectives. Washington: Hemisphere Publishing Corporation.

[ref45] Suls J. M. Wills T. A. (1991). Social Comparison: Contemporary Theory and Research. NJ, Hillsdale: Lawrence Erlbaum Associates.

[ref46] Tao T. Wyer R. S. Zheng Y. (2017). The role of categorization and scale endpoint comparisons in numerical information processing: A two-process model. J. Exp. Psychol. Gen. 146, 409–427. doi: 10.1037/xge0000266, PMID: 28253010

[ref47] Taylor S. E. Buunk B. P. Aspinwall L. G. (1990). Social comparison, stress, and coping. Personal. Soc. Psychol. Bull. 16, 74–89. doi: 10.1177/0146167290161006

[ref48] Thaler R. H. (1999). Mental accounting matters. J. Behav. Decis. Making 12, 183–206. doi: 10.1002/(SICI)1099-0771(199909)12:3<183::AID-BDM318>3.0.CO;2-F

[ref49] Thomas M. Simon D. H. Kadiyali V. (2010). The price precision effect: evidence from laboratory and market data. Market. Sci. 29, 175–190. doi: 10.1287/mksc.1090.0512

[ref50] Wadhwa M. Zhang K. (2015). This number just feels right: The impact of roundedness of price numbers on product evaluations. J. Consum. Res. 41, 1172–1185. doi: 10.1086/678484

[ref51] Wadhwa M. Zhang K. (2019). When numbers make you feel: impact of round vs. precise numbers on preventive health behaviors. Organ. Behav. Hum. Dec. 150, 101–111. doi: 10.1016/j.obhdp.2018.08.005

[ref52] Wang D. Zhu L. Maguire P. Liu Y. Pang K. Li Z.. (2016). The influence of social comparison and peer group size on risky decision-making. Front. Psychol. 7:1232. doi: 10.3389/fpsyg.2016.01232, PMID: 27582723 PMC4987381

[ref53] Wayment H. A. Taylor S. E. (1995). Self-evaluation processes: motives, information use, and self-esteem. J. Pers. 63, 729–757. doi: 10.1111/j.1467-6494.1995.tb00315.x, PMID: 8531044

[ref54] Wegner D. M. Giuliano T. (1980). Arousal-induced attention to self. J. Pers. Soc. Psychol. 38, 719–726. doi: 10.1037/0022-3514.38.5.719

[ref55] Wilson A. E. Ross M. (2000). The frequency of temporal-self and social comparisons in people's personal appraisals. J. Pers. Soc. Psychol. 78, 928–942. doi: 10.1037/0022-3514.78.5.928, PMID: 10821199

[ref56] Wilson A. E. Ross M. (2001). From chump to champ: people's appraisals of their earlier and present selves. J. Pers. Soc. Psychol. 80, 572–584. doi: 10.1037/0022-3514.80.4.572, PMID: 11316222

[ref57] Wisniewski B. Zierer K. Hattie J. (2020). The power of feedback revisited: a meta-analysis of educational feedback research. Front. Psychol. 10:3087. doi: 10.3389/fpsyg.2019.03087 32038429 PMC6987456

[ref58] Xie G. X. Kronrod A. (2012). Is the devil in the details? The signaling effect of numerical precision in environmental advertising claims. J. Advertising 41, 103–117. doi: 10.1080/00913367.2012.10672460

[ref59] Yan D. Pena-Marin J. (2017). Round off the bargaining: The effects of offer roundness on willingness to accept. J. Consum. Res. 44, 381–395. doi: 10.1093/jcr/ucx046

[ref60] Ye J. Zhou K. Chen R. (2021). Numerical or verbal information: The effect of comparative information in social comparison on prosocial behavior. J. Bus. Res. 124, 198–211. doi: 10.1016/j.jbusres.2020.11.053

[ref61] Zell E. Alicke M. D. (2009). Self-evaluative effects of temporal and social comparison. J. Exp. Soc. Psychol. 45, 223–227. doi: 10.1016/j.jesp.2008.09.007

[ref62] Zell E. Alicke M. D. (2010). Comparisons over time: temporal trajectories, social comparison, and self-evaluation. Eur. J. Soc. Psychol. 40, 375–382. doi: 10.1002/ejsp.737

[ref63] Zell E. Strickhouser J. E. (2020). Comparisons across dimensions, people, and time: On the primacy of social comparison in self-evaluations. Soc. Psychol. Personal. Sci. 11, 791–800. doi: 10.1177/1948550619884564

[ref64] Zhang Y. C. Schwarz N. (2012). How and why one year differs from 365 days: A conversational logic analysis of inferences from the granularity of quantitative expressions. J. Consum. Res. 39, 248–259. doi: 10.1086/662612

[ref65] Zhang Y. C. Schwarz N. (2013). The power of precise numbers: a conversational logic analysis. J. Exp. Soc. Psychol. 49, 944–946. doi: 10.1016/j.jesp.2013.04.002

